# The Effects of Hand Massage on Stress and Agitation Among People with Dementia in a Hospital Setting: A Pilot Study

**DOI:** 10.1007/s10484-018-9416-2

**Published:** 2018-09-12

**Authors:** Corinne Schaub, Armin Von Gunten, Diane Morin, Pascal Wild, Patrick Gomez, Julius Popp

**Affiliations:** 1 School of Health Sciences (HESAV), University of Applied Sciences and Arts Western Switzerland (HES-SO), Avenue de Beaumont 21, 1011 Lausanne, Switzerland; 2 Department of Psychiatry, Lausanne University Hospital (SUPAA, CHUV, FBM), Route du Mont, 1008 Prilly, Switzerland; 3 Faculty of Nursing Sciences, Université Laval, Québec, Canada; 4 Institute of Higher Education and Research in Health (IUFRS), University of Lausanne, Quartier UNIL-Epalinges, Biopole 2, Route de la Corniche 10, 1010 Lausanne, Switzerland; 5 Institute for Work and Health, , University of Lausanne and University of Geneva, Lausanne, Quartier UNIL-Epalinges, Biopole 1, Route de la Corniche 2, 1066 Epalinges, Switzerland; 6 INRS, Rue du Morvan, CS 60027, 54519 Vandoeuvre Les Nancy Cedex, France; 7 Department of Mental Health and Psychiatry, Geneva University Hospitals, Geneva, Switzerland

**Keywords:** Dementia, Agitation, Stress, Hand massage, Salivary biomarkers

## Abstract

Agitation in people with dementia is a growing concern as it causes distress for both patients and their nurses and may contribute to relational disorders. Previous studies involving patients with dementia living in long-term care facilities have reported decreased agitation following massage. The objective of this pilot study was to investigate the effect of hand massage on agitation and biological markers of stress in patients with dementia hospitalized in an acute geriatric psychiatry service. In this randomized controlled trial we included 40 agitated patients with dementia with an intervention group and a control group. The study is designed to test the effect of seven hand massages over three continuous weeks on agitation and levels of salivary cortisol (sC) and alpha-amylase (sAA). Compared to the control group, the intervention group exhibited larger increases in sC and sAA at week 1 from before to after the massage, but larger decreases at week 2 and 3, with a significant group effect for sAA at week 2. Agitation scores were not significantly different between the groups but tended to decrease more in the intervention group than the control group. This study provides first encouraging results suggesting that hand massage might have beneficial effects on stress and agitation in hospitalized patients with dementia. It also highlights the challenges associated with conducting such studies with this complex patient population. Further studies are needed to confirm these findings and the benefits of hand massage as part of routine care for patients with dementia.

## Background

Currently, 46.8 million people worldwide has a diagnosis of dementia, and this number will rise to 74.7 million by 2030 (Prince et al. 2015). Along with functional decline, patients with dementia may suffer from communication disorders associated with behavioral and psychiatric symptoms (Van Dyke et al. 2013). Agitation is consensually defined as inappropriate physical or verbal behavior by an individual that is not judged to be the direct result of an unmet need or confusion (Cohen-Mansfield and Billig 1986), and emotional distress has been added to this definition (Cummings et al. 2015). Agitation is a frequent symptom of dementia, affecting up to 85% of people with dementia over a 5-year period (Steinberg et al. 2008). It is a particularly unpleasant symptom for the patient and is strongly related to discomfort (Cohen-Mansfield et al. 2015). Agitation can also cause physical and emotional strain and even feelings of helplessness among nurses caring for patients with dementia (Cerejeira et al. 2012). Many experts recommend prioritizing non-pharmacological interventions to decrease agitation in patients with dementia (Cohen-Mansfield 2015; Von Gunten et al. 2008), and systematic reviews have demonstrated that sensory approaches such as touch and massage have moderate to high efficiency in managing/reducing agitation in this population (Wu et al. 2017). However, these authors agree that many studies investigating the effects of sensory approaches on agitation exhibit methodological weaknesses and that there is still a need for scientific development.

Campbell describes massage as the manipulations of the soft tissue of the body by a therapist or a nurse trained in these techniques (Campbell 2005). Massage in nursing care is considered to include both the comfort and affective touch dimensions (Connor and Howett 2009). While tactile sensitivity decreases with age (Stevens and Choo 1996), sensitivity to affective touch increases (Sehlstedt et al. 2016) and contributes to stress reduction, especially when carried out by a close relative (Ditzen et al. 2007). Affective touch induces physiological effects as it activates areas of the brain involved with emotions and social relationships (Bjornsdotter et al. 2014). Psychological studies indicate that touch induces multiple emotions that are decoded by the person being touched even without seeing the tactile stimulation (Hertenstein et al. 2009). Because of the relative preservation of their limbic systems (Fujii et al. 2014), it appears plausible that people with dementia retain the ability to discern the positive and caring intentions in physical contact such as touch and massage.

Both salivary cortisol (sC) and salivary alpha-amylase (sAA) are considered sensitive biological markers of stress. Cortisol is a neuroendocrine indicator of hypothalamic–pituitary–adrenal (HPA) axis activity (Weibel 2003). SAA is described as a biomarker associated with the activity of the sympathetic nervous system (SNS) and is considered an appropriate measure of change in stress levels (Nater and Rohleder 2009). SAA may also be an indicator of autonomic nervous system (ANS) dysregulation in anxiety-related disorders (Schumacher et al. 2013). SC and sAA have opposite circadian cycles, with sC decreasing during the day and increasing during the night and sAA increasing during the day and decreasing during the night (Nater et al. 2007; Strahler et al. 2010). In response to acute stress, sAA increase is known to precede cortisol release by 13.5 min, and it has been suggested that there is a reliable association between sAA and sC responses at various time lags throughout a stressful situation (Engert et al. 2011). Thus, the concomitant evaluation of the ANS axis with sAA complements sC data and may provide a better understanding of the degree of physiological stress (Engert et al. 2011).

High cortisol levels are part of HPA axis dysregulation in subjects with Alzheimer’s disease (Popp et al. 2015). Additionally, elevated cortisol levels appear to correlate with the rapid progression of dementia (Csernansky et al. 2006) and may cause hippocampal damage (Miller and O’Callaghan 2005).

The positive effects of massage on agitated patients with dementia could also be explained by stress reduction related to decreased anxiety and emotional distress (Cummings et al. 2015; Wu et al. 2017). Anxiety leads to increased levels of stress biomarkers (Chaudieu et al. 2008) and according to some authors, stress precedes agitation behaviors in people with dementia (Smith et al. 2004).

According to literature (Field 2014), the stimulation of cutaneous mechanoreceptors using moderate pressure or stretching appears to mobilize various stress modulation mechanisms that can decrease cortisol levels, among other benefits. Mechanoreceptors are particularly prevalent in the hands and feet (Kennedy and Inglis 2002). A quasi-experimental study found that foot massage has a significant effect on decreasing salivary cortisol (sC) levels, lessening pain and improving mood in aged cancer survivor patients with moderate cognitive impairment (Hodgson and Lafferty 2012). A pilot study involving healthy individuals reported that a 45-min massage at least twice a week favorably decreased sC and increased oxytocin levels (Rapaport et al. 2012). The impact of massage appears to be more significant than that of simple touch when conducted twice a week—even though both approaches appear to have significant effects on biological markers (Rapaport et al. 2012). A recent study assessed a single hand massage intervention with high self-critical individuals with normal cognition and noted a significant decrease of sC 10 min after the end of the intervention and no significant difference for sAA (Maratos et al. 2017). However, the effects of massage on cortisol levels remain controversial (Moyer et al. 2011).

## Aims

To our knowledge, there have been no studies of the effect of hand massage on indices of HPA axis and ANS activity and agitation of cognitively impaired patients hospitalized in an acute geriatric psychiatry service. The present research constitutes the quantitative part of a mixed-methods pilot study. Its objective was to measure the psychophysiological effects of a nursing intervention consisting of performing a series of hand massages over a 3-week period on agitated patients with dementia. More specifically, the first aim was to measure and compare changes in sC and sAA pre- and post-hand massage in the intervention group receiving the hand massage intervention and a control group receiving no hand massages. The second aim was to measure and compare changes in agitation pre- and post-hand massage in the two groups. We postulated that repeated positive hand massages performed by nurses caring for the participants would induce a decrease in the patients’ concentrations of sC and sAA and an improvement in their agitation behaviors.

## Methodology

### Population, Sampling and Setting

The randomized trial included 40 hospitalized older patients (65 years and older) suffering from dementia and agitation. Table 1 provides further demographic and health-related characteristics of the sample.

**Table 1:** Demographic and health-related characteristics of the sample at baseline (T0)

Characteristics	Intervention (n = 20)	Control (n = 20)	*p*-Values
Gender (n; %)			
Male	9 (45%)	11 (55%)	
Female	11 (55%)	9 (45%)	*p* = 0.52^§^
Age (years: M; SD)	81.15 (12.75)	83.14 (4.72)	*p* = 0.69^**∫**^
Length of hospital stay (days: M; SD)	25.00 (26.24)	31.76 (22.99)	*p* = 0.25^**∫**^
CDR scores (n)			
Score = 1	1	6	
Score = 2	16	9	
Score = 3	3	5	*p* = 0.05^**♯**^
HoNOS65+ score (n)			
Score = 1	1	2	
Score = 2	5	4	
Score = 3	8	11	
Score = 4	6	3	*p* = 0.67^**♯**^
Medication (n)			
Analgesics	11	10	*p* = 0.63^§^
Hypnotics	13	15	*p* = 0.65^§^
Antidepressants	10	14	*p* = 0.28^§^
Neuroleptics	18	13	*p* = 0.07^**♯**^

Tests applied: §: Pearson chi^2^ **∫**: Wilcoxon **♯**: Fischer exact

The study also involved 11 nurses and 3 care assistants who volunteered to participate in the research. The study was conducted in a specialized geriatric psychiatry service of a university hospital, and the data were collected between April 2014 and February 2015.

#### Inclusion criteria

##### Patients

All patients hospitalized in the service who suffered from cognitive impairment were considered eligible for the study if they had a minimum score of 1 on the French version of the Clinical Dementia Rating (CDR) (possible range 0–3). The CDR scale is used to measure cognitive and functional performance according to six domains in persons with Alzheimer-type dementia or related dementias: memory, orientation, judgment and problem solving, community affairs, home and hobbies, and personal care (Hughes et al. 1982). Regarding agitation, patients were required to score a minimum of 1 for behavioral disorders (hyperactive, aggressive, disruptive or agitated, uncooperative or defiant) on the French version of the Health of the Nation Outcome Scales 65+ (HoNOS65+) (Canuto et al. 2007). This scale provides an overview of clinical and social problems (behavioral disorders, impairment, symptomatic problems, social disorders). Both instruments are commonly used with clinical populations. These evaluations were conducted by trained psychiatrists not involved in the present study. To be included, patients had to have been admitted to the hospital less than 2 weeks before enrollment in the study and could not be at risk for transfer to another psychiatric service.

##### Nurses

All the nurses and care assistants involved in the study had received specialized training to care for geriatric patients with dementia and had been working in the service for a minimum of 6 months. We considered 6 months the minimum duration for the nurses to feel at ease in the relational contacts with this population.

#### Exclusion Criteria

The following exclusion criteria were applied only to the patients: major known difficulties with body contact, severe psychical decompensation requiring minimal physical stimulation as determined by the medical team, or wounded hands prohibiting the performance of hand massage.

#### Sample Size

Given the pilot nature of the study, 40 patients were recruited. The number of patients was thus above the 12 subjects per group recommended by Van Belle (Van Belle 2002) as a minimum sample size for pilot studies.

#### Randomization

The random allocation sequence—20 patients in the intervention group and 20 patients in the control group—was performed using an independent computerized process with a block randomization size of four. This type of randomization was selected because it is considered to be a technique that achieves balance in the allocation of participants.

#### Blinding

All salivary specimens were collected, coded, stored and sent to the Clemens Kirschbaum laboratory in Dresden, Germany, for blind analysis.

### Intervention

#### Pre-intervention

The hand massage protocol followed a structured plan that could be adapted according to the behaviors and wishes of the patients (massage duration and pressure levels) (Kilstoff and Chenoweth 1998; Remington 2002). The nurses were required to adhere to six steps: (1) ensure they had visual and verbal contact with the patient, ensure the patient had a comfortable and suitable environment (bed, armchair), and face the participants or sit slightly to one side; (2) engage in enveloping contact on the whole hand and the forearm without cream using rhythmic and circular movements; (3) apply hand cream (2 g) to the patient’s forearm, wrist, palm and back of hand, and fingers using the same movements; (4) massage the forearm with moderate pressure, use light gestures on the wrist and the back of the hand, and use moderate pressure on the palm and fingers; (5) end the massage by gradually slowing the manipulations after 8–10 min of massage; and (6) perform steps 2–5 on the second hand. During the entire massage, the nurses observed the behavior of the patient and adapted the pressure and the duration of the manipulations according to their observations (Kilstoff and Chenoweth 1998; Remington 2002). All the participating nurses were trained in the hand massage protocol by the first author who is certified in massage (Joël Savatofsky Training Institute, Dijon, France). The training consisted for all nurses in a 2-h group session during which they carried out the whole procedure while discussing how to adapt it to the patients according to their mood and reactions. The nurses had in their unit a written description of the protocol of the hand massage as a reference. The investigators were in the unit on the days of the measurements and could if necessary answer nurses’ questions.

#### Intervention

##### Intervention group

Based on a preliminary study involving healthy subjects (Rapaport et al. 2012), we decided to have the nurses perform seven massages on each patient over a period of 3 weeks. Our decision took into account both the cognitive and behavioral states of the patients and the availability of the nurses for this study. Seven massages appeared to be sufficient to observe the effects of hand massage on biomarkers in healthy young adults (Rapaport et al. 2012). Salivary samples and agitation assessments were collected on the days of the first (week 1, T1), fourth (week 2, T2), and seventh massage (week 3, T3). On these days, the massages were performed in the unit at 2 pm once from Monday to Thursday according to each patient’s schedule and were conducted in each patient’s preferred location, thereby ensuring a quiet and relaxed environment. This time point (2 pm) was selected because it corresponds to a time during which the unit is relatively quiet as patients are resting and available for social interaction. This time is typically devoted to administrative tasks or medication preparation and was chosen in consultation with the nurses. The time was also ideal because it was placed between scheduled medication administrations—thus avoiding the interference of the direct effects of medication—and positioned between lunch and dinner, which provided good conditions for saliva collection. The required duration of each massage was 16–20 min (minimum of 8 min for each hand); however, the massage was discontinued if the patient indicated a desire to stop. Because of the study’s complex real-life context, it was not possible for each patient to be massaged by the same nurse throughout the entire 3-week intervention period.

If another treatment was planned (physiotherapy, discussion with a social worker or another meeting), the hand massage was performed first. The remaining hand massages, which were performed without saliva collection, were conducted on days chosen by the nurses at a suitable time for both the patient and the nurse.

##### Control Group

The control group patients received usual care. They occasionally participated in leisure activities (drawing group, hairdresser or family visits), received a treatment (such as physiotherapy) or attended a scheduled appointment (in neuropsychiatry, for example). In these situations, saliva collection and CMAI assessments were conducted during the treatment or the appointment once per week for 3 weeks, as for the intervention group.

### Study Process and Measures

#### Level of Agitation

**Fig. 1:**
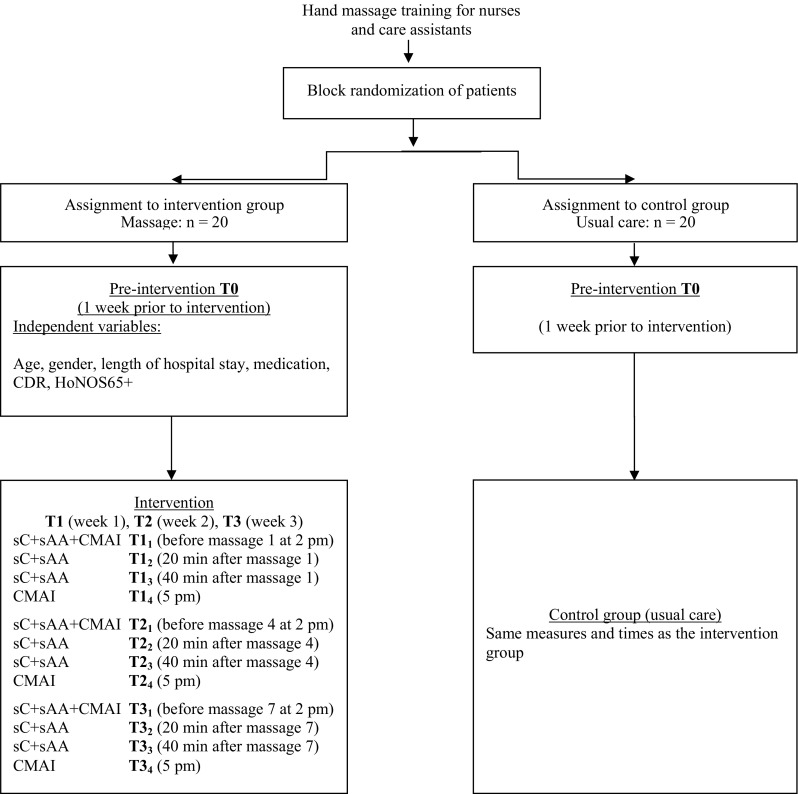
Study process

The agitation measures were collected at 2 pm (before massage) and at 5 pm during an observation period of 10 min in accordance with the recommendations of the Cohen-Mansfield Agitation Inventory (CMAI) (Cohen-Mansfield 1991). Agitation was measured during the intervention at T1 (first massage, first week), T2 (fourth massage, second week), and at T3 (seventh massage, third week) (Fig. 1). Agitation was measured for 10 min at each time point using the CMAI, which is a 29-item rating scale completed by a nurse. The CMAI was developed for use in long-term institutions to retrospectively record the frequency of agitated behaviors over a 2-week period (Cohen-Mansfield 1991). This scale can be used for both long-term observation and observations of a few minutes. The inter-rater reliability of the CMAI has been reported to range from 0.82 to 0.92 (Cohen-Mansfield 1991). A score of “0” indicated that a behavior was not present, “1” indicated that a behavior occurred only once during the observation period, and “2” indicated that a behavior occurred two times, etc. In this study, we counted continued behavior such as “walking” or “making strange noises” as “1” per minute. The total agitation score was calculated by totaling the scores for the individual behaviors (Remington 2002). We also used the following subcategories: verbally non-aggressive (VN) behaviors, verbally aggressive (VA) behaviors, physically non-aggressive (PN) behaviors, and physically aggressive (PA) behaviors (Cohen-Mansfield 2008). This structure, while currently not considered fully validated, is regarded as useful and has been reported previously (Landreville et al. 2007). The validity of the French translation of the CMAI scale has been demonstrated with an inter-rater reliability score of r = 0.72 and an internal consistency of α = 0.77 (Deslauriers et al. 2001). These measures were carried out by two research team members with an inter-rater reliability score of r = 1.00.

#### Salivary Cortisol (sC) and Alpha-amylase (sAA)

We assessed concentrations of sC and sAA from saliva samples collected during the 3-week intervention (T1, T2, T3) at three prescribed times, i.e., 2 p.m., 2.20 p.m., and 2.40 p.m. This allowed us to control for the circadian rhythm of sC and sAA (Nater et al. 2007; Rohleder and Nater 2009) and to optimize comparison between the groups. Saliva samples were collected using a Salivette device (Sarstedt), which involved a cotton swab being placed under the tongue of the participant for 1 min with the assistance of the researchers. To limit potential influences of the environment and the patients’ individual characteristics on the validity of the measures, rigorous control techniques were performed (i.e., the patients were not allowed to smoke, eat, or drink for 1 h before saliva collection, and the samples were frozen at − 20 °C until analysis) (Weibel 2003). Despite these precautions, some of the patients did not have sufficient saliva for analysis, even when the Salivette was left in the mouth for longer than the minimum time required by the procedure. After thawing, the Salivettes were centrifuged at 3000 rpm for 5 min, providing a clear supernatant of low viscosity. Saliva concentrations were measured using commercially available chemiluminescence immunoassay with high sensitivity (IBL International, Hamburg, Germany). The intra- and inter-assay coefficients for sC were below 8%.

#### Demographic and Health Measures

##### Patients

The collected variables included age, gender, length of hospital stay, cognitive impairment measured by the CDR, agitation measured by item no. 1 in the HoNOS65+, and prescribed medication (analgesics, hypnotics, antidepressants and neuroleptics).

##### Nursing Staff Responsible for the Massages

The nursing staff who performed the intervention had an average of 7.1 years (SD = 6.5) of experience caring for patients with dementia (minimum of 2 years) and an average of 5.7 years (SD = 5.8) of experience in the hospital unit (minimum of 1 year). They were mainly women (seven—63%), and their average age was 34 years (SD = 11).

### Statistical Analyses

The demographic and health variables at baseline were calculated and compared between the groups using Wilcoxon, Pearson Chi square, and Fisher’s exact tests. SC, sAA, and agitation were analyzed using an intention-to-treat model. The analyses included all the participating patients who received at least one massage or one control measure. SC, sAA, and total CMAI scores were analyzed using three-level, linear mixed-effects models for longitudinal data, with random intercepts for subject ID and week within subject ID. The effects of the following independent variables, considered fixed effects, were estimated for the different outcome variables: main effects of the intervention (i.e., intervention vs. control), week (T1 vs. T2 vs. T3), and time of day (sC and sAA: 2 p.m. vs. 2.20 p.m. vs. 2.40 p.m.; CMAI: 2 p.m. vs. 5 p.m.) and their 2-way and 3-way interactions. The values of sC and sAA were log-transformed before the statistical analysis to obtain more symmetrical residual distributions. Finally, the linear mixed-model analyses were complemented by independent sample Student’s *t* tests performed on the change scores for the dependent variables between the different times (of day) for each week. These tests allowed us to better interpret the effects (or lack thereof) observed in the linear mixed-model analyses. As a measure of effect size, we report Cohen’s *d*, which reveals the magnitude of the difference between the groups and is particularly useful for small groups because it does not depend on sample sizes (Sullivan and Feinn 2012). Effect sizes are interpreted as small if *d* = 0.20, moderate if *d* = 0.50, and large if *d* = 0.80 (Cohen 1988). All the analyses were performed using Stata 14 (StataCorp LP, College Station, TX). A bilateral alpha level of 0.05 was used for all the tests.

## Results

### Final Sample

**Fig. 2:**
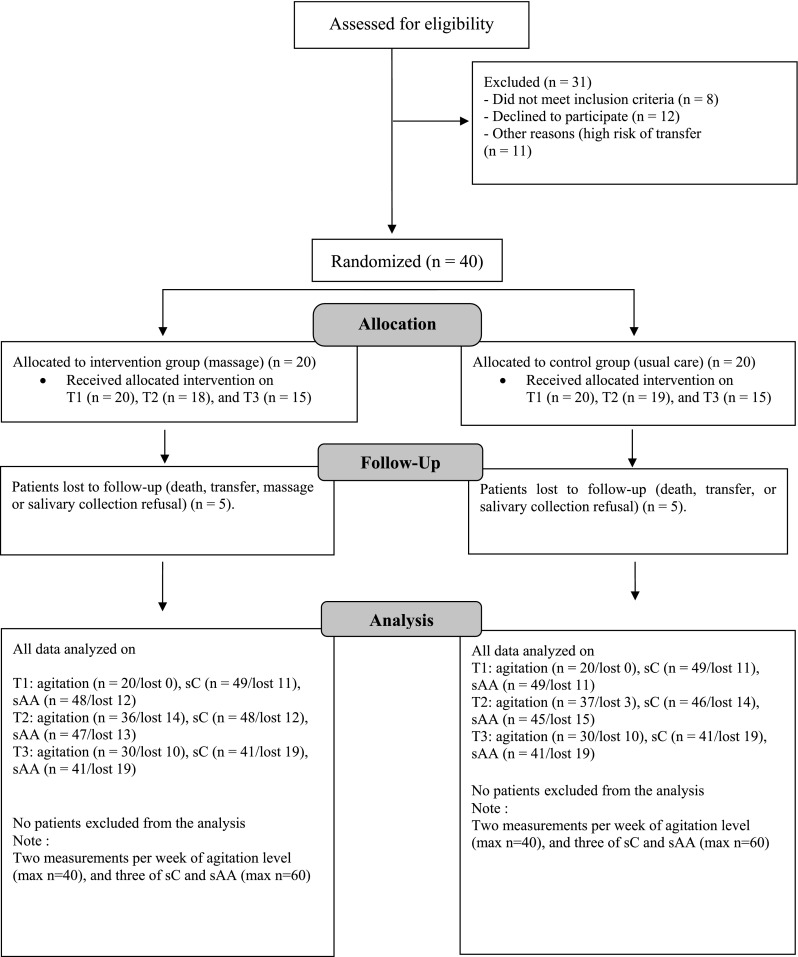
Enrollment and follow up

During the study period, 71 patients were considered for inclusion. Of these, 31 were excluded because they did not meet the inclusion criteria (n = 19) or declined to participate (n = 12). A final sample of 40 patients was block-randomized into the intervention (n = 20) or control (n = 20) groups. During the study, ten patients dropped out because of massage or saliva collection refusal, death or transfer to another hospital. Dropouts were equally distributed in the intervention and control groups (n = 5 in each group). Additionally, it is worth noting that because a strict saliva collection protocol was followed, some samples contained insufficient volume and therefore were not analyzed (see Fig. 2 for details).

### Baseline Results: Independent Variables

At baseline (T0), the distribution of men and women between groups was equal. The groups did not significantly differ in terms of age, length of hospital stay, agitation score (HoNOS65+), or medication. Only the cognitive impairment score (CDR) exhibited a nearly significant difference between the groups (Table 1).

### Salivary Cortisol (sC)

**Fig. 3:**
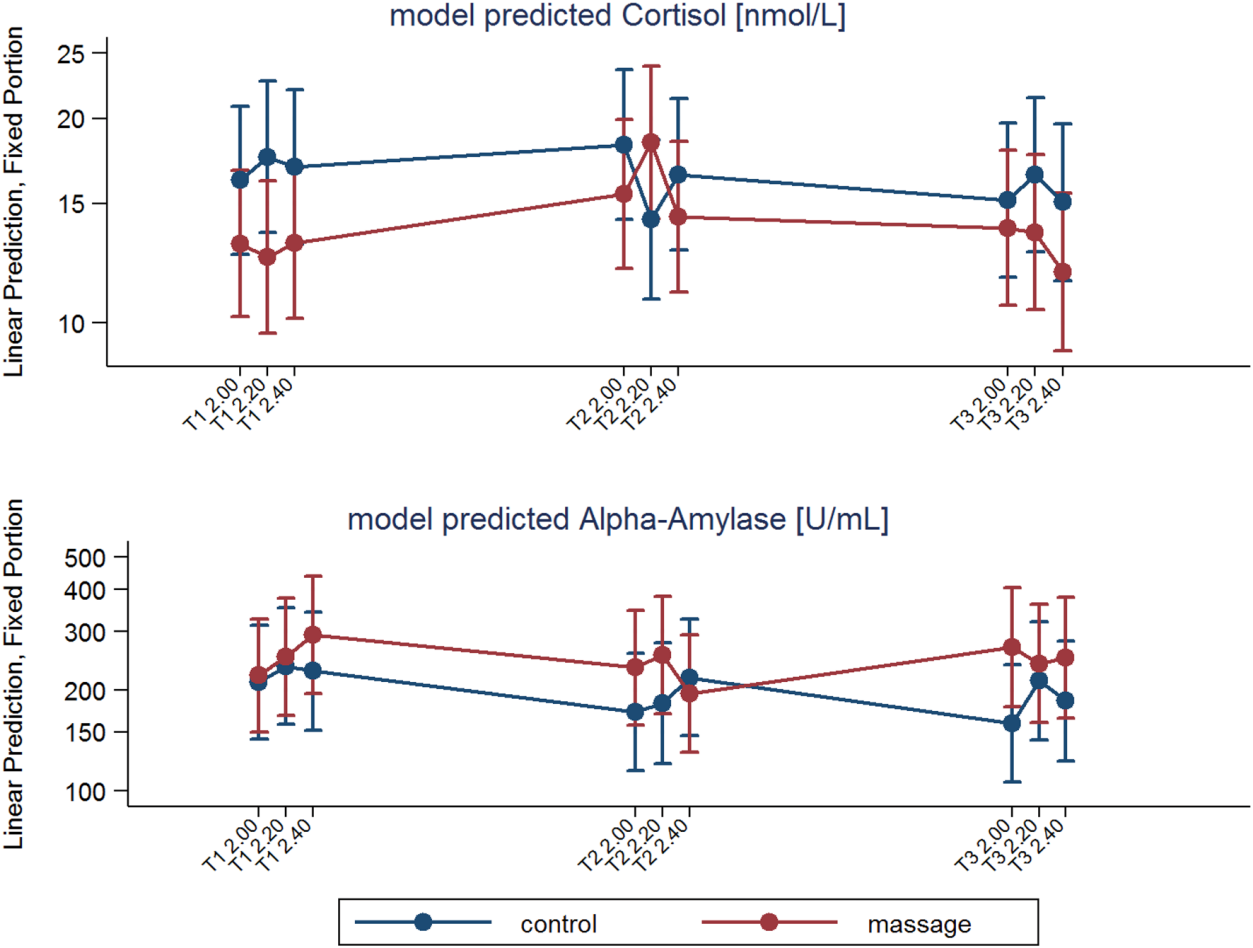
Estimated models for salivary cortisol and alpha-amylase. There are three measures for each week (T1, T2, and T3), i.e.: T1 2.00 = Week 1 at 2 p.m., T2 2.20 = Week 2 at 2.20 p.m., T3 2.40 = Week 3 at 2.40 p.m.

Means and *SD*s of sC are given in Table 2. The estimated model for sC is presented in Fig. 3. A statistically significant three-way interaction between intervention, week, and time of day was observed (*p* = 0.01).

**Table 2:** Means (SDs) of sC, sAA, and CMAI for the intervention and control groups

	sC (nmol/L)		sAA (U/mL)		CMAI	
	Intervention	Control	Intervention	Control	Intervention	Control
Week T1						
T1_1_	16.74 (11.31)	17.55 (6.58)	278.68 (167.05)	304.03 (418.02)	3.5 (4.9)	2.3 (3.3)
T1_2_	14.00 (7.73)	18.42 (7.16)	292.92 (178.66)	279.76 (221.18)		
T1_3_	15.85 (6.41)	18.05 (7.25)	335.91 (202.88)	308.43 (330.10)		
T1_4_					3.4 (4.5)	4.3 (4.2)
Week T2						
T2_1_	14.00 (7.73)	18.78 (6.79)	287.13 (168.19)	286.89 (376.62)	4.2 (4.8)	2.6 (4.2)
T2_2_	21.19 (12.24)	15.17 (5.57)	292.18 (168.94)	317.84 (326.83)		
T2_3_	15.85 (6.41)	17.04 (7.08)	229.96 (138.47)	345.07 (363.90)		
T2_4_					3.9 (4.9)	4.5 (4.3)
Week T3						
T3_1_	15.54 (9.74)	17.72 (9.18)	356.10 (205.46)	234.26 (184.64)	3.1 (3.8)	3.2 (4.0)
T3_2_	14.75 (7.98)	18.83 (9.20)	345.80 (199.47)	267.43 (190.67)		
T3_3_	12.89 (7.06)	16.32 (7.98)	291.80 (114.59)	251.15 (144.38)		
T3_4_					4.6 (5.9)	4.0 (4.3)

*sC* salivary cortisol, *sAA* salivary alpha-amylase, *CMAI* total agitation. T1_1_ = 2 p.m.; T1_2_ = 2.20 p.m., T1_3_ = 2.40 p.m., T1_4_ = 5 p.m. (same for each week)

The results of the *t*-tests performed on the sC change scores are presented in Table 3. During week 1, the group effects were small and not significant; however, during week 2, the sC response pattern was different between the groups. From T2_1_ to T2_2,_ sC increased in the intervention group and decreased in the control group, and the group difference, although not statistically significant, was moderate in size. From T2_2_ to T2_3,_ sC decreased in the intervention group and increased in the control group, and the group difference was significant and large. Globally from T2_1_ to T2_3,_ sC decreased in both groups without group difference. During week 3, sC decreased from T3_1_ to T3_2_ in the intervention group and increased in the control group, and the group difference was small. From T3_2_ to T3_3_, sC decreased in both the intervention and control groups, and the group difference remained small. Globally from T1_1_ to T1_3,_ sC decreased slightly more in the intervention group than in the control group (small to moderate effect).

**Table 3:** Means (SDs) of change scores for log-transformed sC and sAA with t-values and ds of the group effect

	sC				sAA			
	Intervention	Control	*t*	*d*	Intervention	Control	*t*	*d*
Week 1 (T1)								
T1_2_ − T1_1_	− 0.006 (0.106)	− 0.007 (0.106)	*t*(28) = 0.03	− 0.01	0.058 (0.195)	0.011 (0.264)	*t*(28) = 0.55	− 0.20
T1_3_ − T1_2_	0.006 (0.063)	− 0.011 (0.109)	*t*(27) = 0.52	− 0.19	0.065 (0.167)	− 0.041 (0.190)	*t*(27) = 1.60	− 0.59
T1_3_ − T1_1_	0.015 (0.126)	0.000 (0.144)	*t*(29) = 0.30	− 0.11	0.123 (0.175)	0.030 (0.288)	*t*(28) = 1.06	− 0.39
Week 2 (T2)								
T2_2_ − T2_1_	0.056 (0.214)	− 0.088 (0.180)	*t*(26) = 1.91	− 0.73	0.039 (0.170)	0.044 (0.311)	*t*(26) = 0.06	0.02
T2_3_ − T2_2_	− 0.099 (0.166)	0.044 (0.146)	*t*(26) = 2.42*	0.92	− 0.114 (0.150)	0.090 (0.207)	*t*(26) = 3.03**	1.13
T2_3_ − T2_1_	− 0.043 (0.114)	− 0.039 (0.110)	*t*(28) = 0.09	0.03	− 0.075 (0.120)	0.110 (0.192)	*t*(28) = 3.15**	1.15
Week 3 (T3)								
T3_2_ − T3_1_	− 0.006 (0.087)	0.017 (0.091)	*t*(25) = 0.68	0.26	− 0.049 (0.322)	0.118 (0.281)	*t*(25) = 1.43	0.55
T3_3_ − T3_2_	− 0.044 (0.070)	− 0.031 (0.126)	*t*(23) = 0.75	0.13	0.038 (0.292)	− 0.062 (0.244)	*t*(23) = − 0.92	− 0.37
T3_3_ − T3_1_	− 0.060 (0.095)	− 0.010 (0.157)	*t*(23) = 0.98	0.39	− 0.021 (0.197)	0.048 (0.246)	*t*(23) = 0.77	0.31

*sC* salivary cortisol, *sAA* salivary alpha-amylase. There are three measures for each week (T1, T2, and T3), i.e.: T1:T1_1_ = 2 pm, T1_2_ = 2.20 pm, T1_3_ = 2.40 pm

*p < 0.05; **p < 0.01

The raw data (Table 2) appear to indicate high sC scores throughout the study. The sC means at 2 pm were more elevated compared to published data on healthy older adults (min 14.00 nmol/L, max 18.78 nmol/L vs. 4 nmol/L to 6 nmol/L (Strahler et al. 2010).

### Salivary Alpha-amylase (sAA)

The estimated model for sAA is presented in Fig. 3. A statistically significant three-way interaction between intervention, week, and time of day was observed (*p* = 0.02).

The results of the *t*-tests performed on the sAA change scores are displayed in Table 3. During week 1, from 2 p.m. to 2.20 p.m. (T1_1_ to T1_2_), sAA increased in both groups with small between-group differences. From 2.20 p.m. to 2.40 p.m. (T1_2_ to T1_3_), sAA increased in the intervention group and decreased in the control group, and the size of this group difference was moderate. Globally from 2 p.m. to 2.40 p.m. (T1_1_ to T1_3_), sAA increased slightly more in the intervention group than in the control group (small to moderate effect). During week 2, sAA increased in both groups similarly from T2_1_ to T2_2_. In contrast to week 1, sAA decreased from T2_2_ to T2_3_ in the intervention group but not in the control group, and the group difference was significant and large. Overall, from T2_1_ to T2_3_, sAA decreased in the intervention group and increased in the control group, and this group difference was also significant and large. During week 3, from T2_1_ to T2_2_, sAA decreased for the first time in the intervention group and increased in the control group, and the group difference, although not statistically significant, was moderate. From T2_2_ to T2_3,_ sAA increased in the intervention group and decreased in the control group, and the group difference was small to moderate. Globally from T1_1_ to T1_3,_ sAA decreased in the intervention group and increased in the control group, and the group difference was small to moderate.

Similar to the results for sC, the raw data (Table 2) appear to indicate high sAA scores throughout the study, with sAA means at 2 pm from min 234.26 U/mL to max 356.10 U/mL compared to a healthy elderly reference population with means from min 150 U/mL to max 220 U/mL (Strahler et al. 2010).

#### Agitation Scores: Cohen-Mansfield Agitation Inventory (CMAI)

The mixed modeling of the CMAI revealed no significant interactions or main effects, except the main effect for time of day, reflecting an overall increase in CMAI between 2 p.m. and 5 p.m. (*p* = 0.02) during the 3 weeks of the intervention. The results of the *t*-tests performed on the agitation change scores are provided in Table 4 and reveal that none of the group comparisons was significant. During week 1, the CMAI scores decreased in the intervention group and increased in the control group from T1_1_ to T1_4_, and the group difference was moderate. All the sub-scores for agitation in the intervention group decreased except PN agitation, while only VN agitation decreased in the control group. For PA agitation, the group difference was moderate and nearly significant (*p* = 0.08). During week 2, CMAI again decreased from T2_1_ to T2_4_ in the intervention group and increased in the control group, and the group difference was small to moderate. The sub-score for PN increased less in the intervention group than in the control group, and the group difference was small. During week 3 (T3_1_ to T3_3_), CMAI increased in both groups with a small group difference. The sub-scores for PN and VA agitation increased in both groups without group differences. The PA agitation sub-score did not change in the intervention group and decreased in the control group, and the group difference was small (Table 4).

**Table 4:** Means (SDs) of change scores for CMAI and subscales of agitation with t-values and ds of the group effect

	Intervention	Control	*t*	*d*
Week 1 (T1_4_ − T1_1_)				
CMAI	− 0.1(4.2)	2.0(4.5)	*t*(38) = 1.55	0.49
PA	− 0.1(0.3)	0.1(0.2)	*t*(38) = 1.76^a^	0.55
PN	0.7(2.8)	1.9(3.1)	*t*(38) = 1.27	0.40
VA	− 0.3(0.6)	0.1(1.9)	*t*(38) = 0.99	0.31
VN	− 0.5(2.3)	− 0.1(2.7)	*t*(38) = 0.42	0.13
Week 2 (T2_4_ − T2_1_)				
CMAI	− 0.3(5.9)	1.7(4.1)	*t*(34) = 1.17	0.39
PA	− 0.1(0.2)	− 0.2(0.6)	*t*(34) = − 1.02	− 0.34
PN	0.2(5.1)	1.3(3.5)	*t*(34) = 0.71	0.23
VA	− 0.2(1.2)	− 0.3(1.3)	*t*(34) = − 0.39	− 0.13
VN	− 0.2(2.8)	1.1(2.3)	*t*(34) = 1.44	0.48
Week 3 (T3_4_ − T3_1_)				
CMAI	1.5(6.1)	0.7(5.6)	*t*(28) = − 0.37	− 0.14
PA	0.0(0.0)	− 0.2(0.7)	*t*(28) = − 1.00	− 0.36
PN	1.3(4.5)	1.1(5.0)	*t*(28) = − 0.15	− 0.05
VA	0.5(2.7)	0.7(1.2)	*t*(28) = − 0.25	0.09
VN	− 0.3(2.3)	− 0.8(2.7)	*t*(28) = − 0.57	− 0.21

*CMAI* total agitation score, *PA* physically aggressive agitation, *PN* physically non-aggressive agitation, *VA* verbally aggressive agitation, *VN* verbally non-aggressive agitation. There are two measures for each week (T1, T2, T3), i.e., T1:T1_1_ = 2 pm, T1_4_ = 5 p.m.

^a^p = 0.08

#### Examination of Independent Variables as Possible Covariates

There was no significant difference between the groups related to medication during the 4 weeks of the study. Age, gender, CDR, HoNOS65+ and duration of massage exhibited no significant effect on sC and sAA after 20 and 40 min. The massage duration ranged from 3 to 25 min with an average of 15.9 min (SD = 4.7).

## Discussion

### Interpretation of the sC and sAA Results

Analyses revealed that the control and intervention groups differed significantly in terms of their salivary cortisol (sC) and salivary alpha-amylase (sAA) patterns across weeks and times of day (i.e., significant three-way interactions). A *post hoc* test of the specific changes between times of day revealed that the main differences between the two groups were found during week 2, when the patients received their fourth massage. In that second week, we observed unique and unexpected increases in sC and sAA at the end of the intervention group’s fourth massage, followed by a significant decrease in both biomarkers 20 min later. Nurses’ notes indicated that during this massage some patients sustained positive verbal interaction, did not want to continue with a long massage, or were a little tense. Three patients showed sexual behaviors which nurses felt were unpleasant. These behaviors were not reported during the massages in weeks 1 and 3.

In the third week, sC and sAA had already simultaneously decreased by the end of the massage. These results revealed the positive effects of hand massage on reducing stress biomarkers, although the group difference did not reach statistical significance. They also highlighted the need for repeated massage sessions before any biological effects could be observed, as reported in the existing literature (Rapaport et al. 2012). These results are in accordance with nurses’ notes, which indicated that patients no longer wanted to speak during the massage and were more relaxed and sleepier than in the two previous weeks. Patients did not show any sexualized behavior.

When looking at the changes in sC and sAA between 2 p.m. to 2.40 p.m., across all 3 weeks, the control group showed smaller mean increases than the intervention group in week 1. This pattern was reversed during weeks 2 and 3, pointing to larger decreases in the intervention group than in the control group. However, the multiple time-lags for sAA and cortisol stress responses (Engert et al. 2011) limit interpretations about the variability of these biomarkers’ results during the intervention. According to Engert and co-authors, the release of alpha-amylase precedes the release of cortisol by 13.5 min in healthy adults. Recent literature suggests that sAA should be collected immediately after the hand massage, and that sC should be collected 10 min later, due to the difference in time needed for sAA and sC to reach their peak levels (Maratos et al. 2017). In the present study, sC and sAA were collected together in one sample at the end of the massage and in another 20 min later. By taking into account the specific timings for the interpretation of sC and sAA data, starting from the intervention’s second week, our results are in agreement with those reported by Maratos et al. (2017), who noted a significant decrease in sC 10 min after the end of the intervention and an insignificant decrease in sAA at the end of the hand massage.

It is noteworthy that our sample’s mean sC and sAA levels exhibited substantial individual variability, with large standard deviations. These results appear to indicate particularly high mean sC and sAA levels compared with those of healthy older adults (Strahler et al. 2010). This could be a sign of elevated stress and may indicate dysregulation of the HPA axis (Ouanes et al. 2017; Popp et al. 2015) and the ANS, (Schumacher et al. 2013) which may alter stress-response in patients with Alzheimer’s disease (Rothman and Mattson 2010). These findings highlight the importance of developing therapeutic tools to reduce stress among vulnerable populations.

Establishing the factors that may differentiate patients responding well to the intervention from those not responding to the intervention was beyond the scope of the present pilot study. As suggested by the authors of recent studies involving healthy populations (Maratos et al. 2017; Marchand et al. 2014, 2016), future studies including larger samples and assessing psychosocial data at each collection of saliva may shed light on the determinants of intra- and inter-patient variations in the levels of sC and sAA among patients with dementia receiving hand massage. These psychosocial data should include measurements adapted to patients with cognitive impairments, such as verbal and nonverbal indicators of discomfort (Stevenson et al. 2006).

### Interpretation of Agitation (CMAI) Results

The analyses revealed that CMAI only exhibited a significant main effect for time of day, reflecting an overall increase in CMAI between 2 p.m. and 5 p.m. This corresponds with the literature indicating an increase in agitation in this population at the end of the afternoon (Khachiyants et al. 2011). Mean agitation was very low for both groups during the study duration, probably because of the use of medication such as antidepressants, analgesics, neuroleptics, and hypnotics—which are frequently prescribed during hospitalization to treat neuropsychiatric symptoms including agitation—and because of other usual psychosocial approaches and nursing care. There was no significant difference in CMAI between the groups during the 3 weeks of the intervention, which could be due to the large time interval between the intervention and the last measurement at 5 pm. This finding contrasts with other studies that have reported a decrease in agitation 1 h after the intervention (Remington 2002; Sandee and Bryn 2008). However, as expected, agitation levels decreased between 2 p.m. and 5 p.m. for the intervention group during the two first weeks of intervention, and the results were in the expected direction. The third week exhibited an increase in the CMAI scores for both groups, particularly for the PN and VA subcategories of the intervention group. In the intervention group, PN behavior was particularly associated with “wandering”. In the unit where the study was conducted, wandering is a behavior that should be interpreted with caution as a form of ‘agitation’. These specific patients suffer from numerous comorbidities and are encouraged to be physically active as soon as they feel better. Physicians also reduce medication once agitation behaviors stabilize; this practice may have encouraged wandering and VA and could explain the slight increase in agitation in both groups. However, these results could also be explained by the loss of participants during the study, the small sample size and the low mean for agitation at 2 pm in both groups.

### Potential Effects of Nurse–Patient Interactions

The nurses reported to the researchers that performing massages at a fixed time (1st, 4th, and 7th massages) was less conducive to a calm and relaxed atmosphere than performing them at a time of day specifically chosen according to each patient’s specific needs and nurses’ availabilities (2nd, 3rd, 5th, and 6th massages). Doing massages at a fixed time in a busy schedule could sometimes have caused nurses to have less patience and empathy. Furthermore, patients did not necessarily always want a massage at the time planned or wanted to chat with the nurse during the massage. Some patients probably also had less personal affinity with certain nurses. These conditions may sometimes have affected both patients’ and nurses’ willingness to take part in a massage, which may have influenced the modifications in the biomarkers. Indeed, a recent study has described how neurobiology shapes affective touch by reviewing the reciprocal influences of gentle touch and contextual information (such as the subject’s touch expectations and motivations) (Ellingsen et al. 2016). In this way, a pleasant experience of touch can turn unpleasant if the toucher’s perceived intentions do not correspond to the recipient’s expectations (Gazzola et al. 2012), and this could influence biomarker levels too. Further studies are needed to explore the influence of caregivers’ emotions and empathy on biomarkers and agitation levels in patients with dementia during hand massage.

### Strengths and Limits

Because of this study’s design, it was not possible for blinded investigators to perform the CMAI scoring. Despite the randomization, the CDR scores were higher in the intervention group, and this difference was nearly significant. It might have been good to include them as predictors in the analyses. The withdrawal of some patients and the loss of salivary data (because of insufficient saliva) reduced the internal validity of the study given the small sample size. Internal validity was also reduced by the variation in massage duration, the fact that all seven massages were not given by the same nurse, and that massages 1, 4, and 7 were at fixed times but massages 2, 3, 5, and 6 were at chosen times. Nevertheless, the external validity of this study can be regarded as good. Clearly, the pragmatic dimension of the intervention aids in assessing the effectiveness of hand massage in real-life, routine, practice conditions and in determining the conditions for using hand massage with vulnerable populations such as agitated patients with dementia. This type of trial produces results with high ecological validity that can be used to formulate routines for practice settings and should guide future research protocols to better adapt to patients’ individual reactions and preferences (Patsopoulos 2011).

## Conclusion

We observed a statistically significant three-way interaction between intervention, week, and time of day for both sC and sAA. These interactions mainly reflected group differences during week 2. In week 2, sC and sAA increased during the massage, but this was followed by significant decreases. In the third week, sC and sAA had already dropped simultaneously by the end of the massage. A deeper exploration of the contextual conditions of nurse–patient interactions during massage is required. Our results also appear to indicate the added value of building environmental and psychosocial models in clinical studies using stress biomarkers, as has been done in other studies with healthy populations (Maratos et al. 2017; Marchand et al. 2014, 2016). Finally, this pilot study demonstrates the need to perform several massages before observing biological modifications in patients with dementia.

This study also suggests that there is a positive effect on agitation 3 h after a hand massage, but surprisingly, the intervention exhibited a greater effect during the two first weeks of the intervention than during the third week. In addition, the results of this study reveal high mean levels of sC and sAA in cognitively impaired patients hospitalized in an acute geriatric psychiatry service. This may suggest high stress levels and/or HPA axis (Popp et al. 2015) and possibly ANS dysregulation (Schumacher et al. 2013) in this population.

Performing hand massage on agitated, cognitively impaired patients, which initially appears to be a simple activity without side effects (Holliday-Welsh et al. 2009; Remington 2002), is nevertheless very complex as it typically involves multiple implicit and automatic cultural and social interpretations (Connor and Howett 2009). Hand massage is a sensitive and intimate relational activity that requires a mutual agreement that is sometimes difficult to obtain from severely cognitively impaired patients. It is necessary to gradually introduce hand massage into routine care according to both the preference and pace of each patient and the availability of nurses. Conformity with the massage protocol and the duration of the massages varied due to the decision to adapt the intervention to patients’ needs. The intervention’s feasibility is good, but it requires the flexibility to be able to postpone a massage in the case of patient refusal. Finally, this research is encouraging for the development of pragmatic studies of non-pharmacological interventions while revealing the limits of randomized experimental studies with complex populations. Other approaches, which more effectively consider the characteristics, preferences, and unpredictability of patients with dementia, such as longitudinal randomized crossover studies or in-depth case studies, should be promoted.
